# Chaihu Longgu Muli Decoction for post-stroke insomnia: A protocol for systematic review and meta-analysis

**DOI:** 10.1097/MD.0000000000033376

**Published:** 2023-04-14

**Authors:** Xuedi Huang, Yue Xiong, Sichen Jiang, Lihua Tang, Xingzhen Lin, Xinyue Fang, Yuzhen Shi, Wanning Lan, Yaying Xie, Tianzhong Peng

**Affiliations:** a Jiangxi Province Hospital of Integrated Chinese and Western Medicine, Jiangxi, China; b Nanchang Hongdu Hospital of Traditional Chinese Medicine, Jiangxi, China; c Guangzhou University of Chinese Medicine, Jiangxi, China.

**Keywords:** Chaihu Longgu Muli Decoction, poststroke insomnia, protocol, systematic review

## Abstract

Poststroke insomnia (PSI) is a frequent complication of stroke usually as a comorbidity of poststroke depression and mainly occurs within the first 6 months after stroke.^[1]^ Addressing PSI to improve stroke prognosis is of great value. Herbal medicine like Chaihu Longgu Muli Decoction (CLMD), which is commonly considered to be a good treatment for depression and epilepsy, has the therapeutic potential on PSI; however, insufficient systematic reviews were conducted to testify its efficacy. Therefore, the objective of this paper is to provide reliable evidence of the efficacy and safety of CLMD on PSI and a foundation for further investigation.

**Methods:**

The literature of clinical randomized controlled trials (RCTs) regarding CLMD for PSI published before June of 2021 will be retrieved in the databases, and 2 investigators will be asked to collect and crosscheck the data independently. For the including studies, the quality evaluation on methodology will be assessed in the light of the Cochrane Handbook for Systematic Review of Interventions V.5.1.0 as well as the quality of evidence will be evaluated by the Grading of Recommendations Assessment, Development, and Evaluation. Besides, the assessment of heterogeneity and reporting bias, the sensitivity analysis and the subgroup analysis will be conducted. Stata 15 will be applied to analyze the above data.

**Results:**

The review will conduct a high-quality synthesis on present evidence of CLMD for PSI.

**Conclusion:**

The conclusion of the study will indicate whether CLMD is effective and safe for PSI.

## 1. Introduction

### 1.1. Description of the condition

Poststroke insomnia (PSI), known to be both a risk factor and complication of stroke, can negatively impact the prognosis and functional recovery after stroke.^[2,3]^ According to the standard diagnostic criteria, insomnia is described as the difficulty in starting or maintaining sleep or early morning awakening which affects daytime functioning. Symptoms above should last over 3 months and at least 3 nights a week.^[4]^ One meta-analysis indicated pooled prevalence of 38.2% with 40.70% of studies using nondiagnostic tools and 32.21% of which using diagnostic assessment tools.^[5]^ Another reported that the incidence of PSI ranged from 25% to 82% which indicates the high variability of the prevalence of PSI.^[6–8]^ For pharmacological therapy, Western medicine has few ideal therapeutic drugs. Benzodiazepines, antidepressants such as mianserin and hypnotics are not recommended, and though Zolpidem can relatively increase brain-derived neurotrophic factor secretion and protect the neurovascular unit in acute stroke, it is related to ischemic stroke risk.^[9]^ On the contrary, there is much room for Traditional Chinese Medicine. The alternatives are in varied forms, such as acupuncture, massage, auricular acupoint, catgut embedment in acupoint, the music of 5 phases, Tai Chi, also the decoction including Si Ni San, Tianma Gouteng Yin, Da Ding Feng Zhu, Gui Pi Decoction, Chaihu Longgu Muli Decoction (CLMD), self-designed Ningxin Anshen Formula and so on.^[10–14]^ Especially, Herbal medicine shows a significant efficacy in relieving insomnia.^[15]^ Other treatments like cognitive-behavioral therapy, bright light therapy are also mentioned.^[16,17]^ Recognized classification or clinical diagnostic criteria like DSM-IV/V, ICSD-2, ICD-10 are applied to assess symptoms of insomnia. Other instruments such as Pittsburgh Sleep Quality Index, Hamilton Depression Scale, Epworth Sleepiness Scale or self-reported insomnia are also involved.^[18]^ In addition, polysomnogram signals and the improved single-channel electroencephalogram signals are used to detect the sleep stages and sleep disorders,^[19]^ and ISI-3 is also applicable as a brief screening tool for insomnia.^[20]^

### 1.2. Description of the intervention

According to the theory of Chinese Medicine, the possible mechanism of PSI can be interpreted as the disorderly qi flowing caused by the liver losing its dominant position of harmonizing qi. The constrained qi transforms into fire and disturb the spirit, which leads to sleep disorder.^[12]^ CLMD, a classic prescription from “Treatise on Febrile Diseases,” is conventionally applied in the situation of fullness in the chest, irritability with occasional attacks of palpitations, delirious speech and other symptoms belong to the property of fire. Nowadays its application has been widen to treat nervous system diseases including PSI by its weighing down and calming the floating spirit. With ingredients of Radix Bupleuri (Chaihu), Radix Scutellariae (Huangqin), Rhizoma Pinelliae Ternatae (Banxia), Radix Ginseng (Renshen), Rhizoma Zingiberis Officinalis Recens (Shengjiang), Ramulus Cinnamomi Cassiae (Guizhi), Sclerotium Poriae Cocos (Fuling), Os Draconis (Longgu), Concha Ostreae (Muli), Radix et Rhizoma Rhei (Dahuang), Fructus Zizyphi Jujubae (Dazao), Minium (Qiandan), CLMD can improve sleep quality and relieve the symptom caused by poor sleep. Hence, this formula may be used in treating such biomedically-defined disorders as neurosis, schizophrenia, depression, hysteria, epilepsy, hypertension, hyperthyroidism, and postconcussion syndrome.^[21–23]^ In terms of recent researches, despite a risk of bias from the studies included, CLMD showed great potential to be an alternative treatment for PSI compared with conventional medications.^[24]^

### 1.3. Possible mechanism of the intervention

According to “Huang Di Nei Jing,” the basic pathogenesis of stroke is the imbalance of yin and yang, ying and wei, the rebellion and the offense of qi and blood, and the invasion of pathogenic factors on the brain. It can be described in detail that with a basic dysfunction of 5 internal organs, “phlegm, fire, stasis, qi, blood” pathological factors are generated. These pathological factors often associate with each other both and above so that the smooth flowing of qi and blood is blocked and the disharmony between yin and yang occurs.^[25]^ It results in the failure of yang to enter yin and internal injury of fatigue by which insomnia is caused. As assistant ingredients, Longgu and Muli is aimed at sleep disorder and profuse dreaming directly. Their properties and actions of mineral substances make floating yang in spirit subdued. Longgu can suppress fright and calm the mind and Muli can calm the liver, enrich yin and extinguish wind so that ying and yang, ying and wei levels can be harmonized. Fuling acts on the hand shaoyin heart channel to nourish the heart and calm the mind. Dahuang disperses stomach heat to address restless sleep caused by stomach disharmony. And Guizhi is discovered to have positive effect on improving insomnia symptoms due to its chemical composition of Cinnamic aldehyde, cinnamic acid, 2-methoxycinnamic acid. Ginseng Saponins which is found the active ingredients of Renshen, can significantly improve SSR and autonomic nerve function in patients with insomnia. Together with Fuling, it can strengthen the Spleen qi and ensure that it is not further injured by the treatment.^[23,26]^ In terms of the modern pharmacology, some researches proposed that the formative mechanism of CLMD is possibly associated with the process of neuronal regeneration and apoptosis, sympathetic hyperactivity and inflammatory reaction, so that regulates the neuroendocrine system centered on hypothalamus pituitary adrenal axis.^[27]^ Another claims that CLMD takes effect by regulating patient neurotransmitter level such as improving serum 5-HT and DA.^[28]^ In short, CLMD could take effect on the treatment for PSI both from the perspective of Chinese medicine and Western medicine.

### 1.4. The importance of this review

Preliminary data suggest a high frequency of PSI and their association with a less favorable stroke outcome, while treatment data are scarce.^[29]^ Currently, there are insufficient systematic reviews of CLMD for PSI, 10 experimental researches in Chinese and 1 meta-analysis in English are found. Furthermore, the dispersed researches, unregistered indicators, lack of key regulatory elements are the potential defects. Therefore, there is a necessity to update and improve the systematic review and meta-analysis of CLMD of PSI to offer high-quality evidence-based evidence and obtain better guide clinical practice. This paper will be performed on the basis of the latest literature from 5 comprehensive databases, in the hope that provide reliable evidence of the efficacy and safety of CLMD on PSI.

## 2. Methods

### 2.1. Materials and methods

The study will keep to the guide from the Preferred Reporting Items for Systematic Reviews and Meta-Analyses and has been registered in PROSPERO (CRD42021252526). The protocol will be structured following the Preferred Reporting Items for Systematic Reviews and Meta-Analyses Protocols guidelines.

### 2.2. Eligibility criteria

#### 2.2.1. Study characteristics.

Except for those references with incomplete or wrong data, all randomized controlled trials (RCTs) will be included, irrespective of publication status. Only studies in Chinese or English will be included.

#### 2.2.2. Object of study.

Taking the recognized classification or clinical diagnostic criteria for insomnia diagnosis, like DSM-IV/V, ICSD-2, ICD-10, and the patients involved suffered from PSI. No age, sex or territory limit. Patients with other diseases combined would also be excluded.

#### 2.2.3. Intervention measure.

The patients in control group receive routine western medicines, while those in treatment group were treated with CLMD. The plus and minus of CLMD must conform to the principles of Monarch, minister, assistant and guide in Traditional Chinese Medicine prescription. The medicine could come in a number of different forms, such as bolus, powder, plaster, pellet, tablet, oral liquid and so on.

### 2.3. Outcome

The primary outcome will be measured by Pittsburgh Sleep Quality Index. The secondary outcomes will be measured by Epworth Sleepiness Scale, Stroke Specific Quality of Life Scale and the safety index.

### 2.4. Search strategy

The following electronic databases will be systematically searched up to June 2021: Cochrane Central Register of Controlled Trials, Embase, PubMed, Web of Science, the Chinese National Knowledge Infrastructure, the Chinese Biomedical Literature Database, the Chinese Scientific Journal Database and the Wanfang Database. The key words for literature searching are “Chaihu Longgu Muli Decoction” and “Post-stroke insomnia.” The search strategy for PubMed is shown in Table 1.

**Table 1:** Search strategy for the PubMed database.

#1 Post-stroke insomnia
#2 Stroke
#3 Insomnia OR wakefulness OR agrypnia
#4 Chaihu longgu muli decoction OR Chaihu Longgu Muli Decoction OR chai hu longgu muli decoction OR Chaihu Longgu Muli Tang OR chaihu longgu muli tang
(#1 OR (#2 AND #3)) AND #4

We would also search additional data through other sources: Hand searching, Conference proceeding, International Clinical Trials Registry Platform and Chinese Clinical Trial Registry.

### 2.5. Study selection

**Figure 1.**
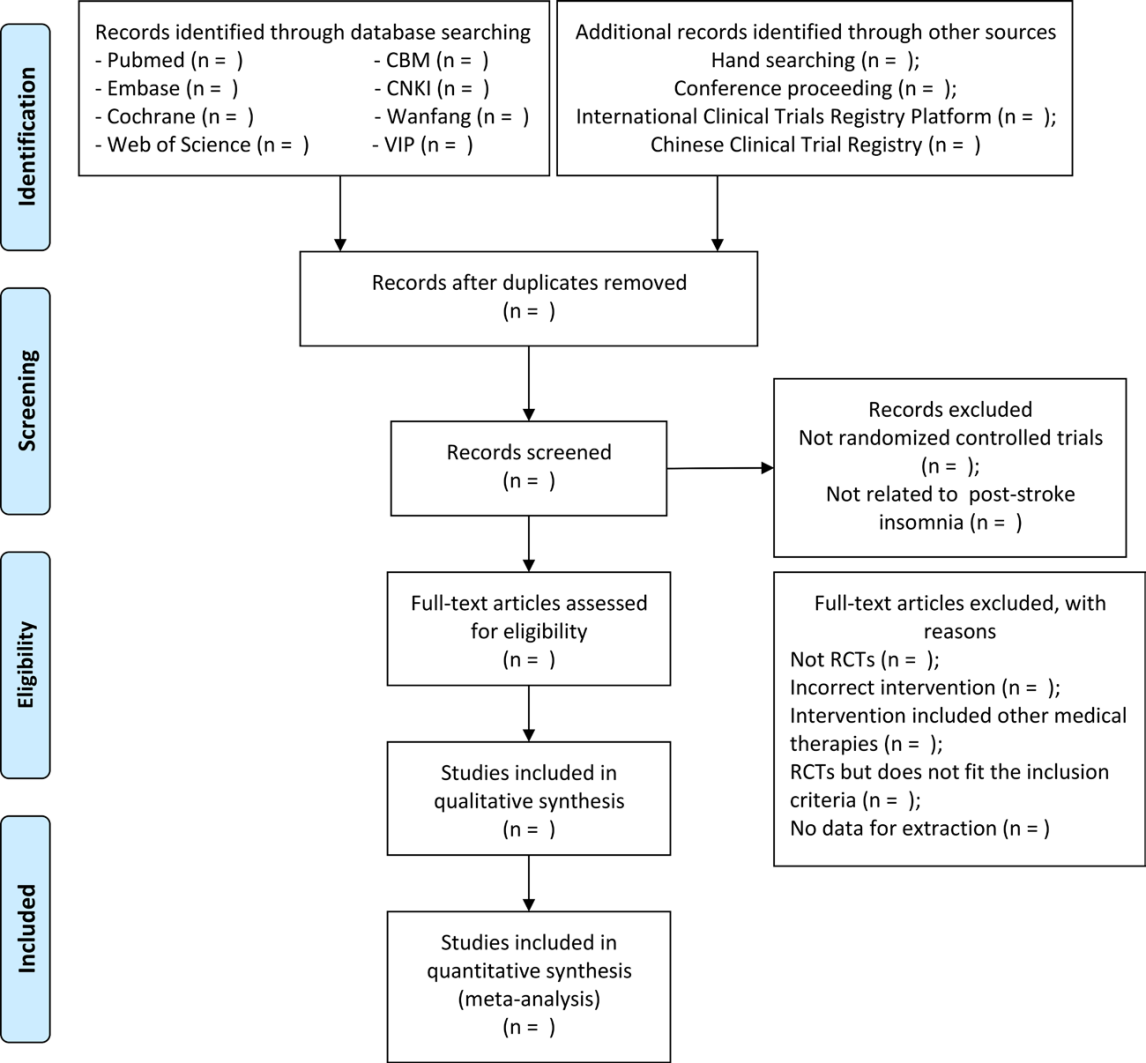
PRISMA flow chart of study selection process. PRISMA = preferred reporting items for systematic reviews and meta-analyses.

To screen all the studies, we will use the EndNote X9 to remove the duplicates. The potentially relevant studies will be first screened by 2 investigators (ZZ and ZC) independently according to the titles and the abstracts above. And the studies will be excluded when the studies are not human studies, not clinical trials or not related to PSI. Then we will read the full articles and the studies assessed as eligible will be included. By reading full text, some studies will be excluded for the following reasons: Not RCTs, Incorrect intervention, Intervention included other medical therapies, RCTs but does not fit the inclusion criteria, No data for extraction. Detailed screening process is shown in Figure 1.

### 2.6. Data collection

Data collection will be completed and crosschecked independently by 2 investigators. The extracted data mainly contain basic information, characteristics of trial subjects, intervention measures, results of the studies. In case of divergence, the third reviewer (GC) should participate in the discussion and help to solve the problems. If the data about the outcomes are insufficient, corresponding author of the study will be contacted by e-mail or telephone. We will only analyze the available data if enough information cannot be acquired in this way. The potential impact of missing data will be considered on the results of meta-analysis.

### 2.7. Quality evaluation on methodology

The risk of bias of the involved studies will be evaluated independently by 2 verifiers (TC and YL) applying the assessment tool based on the standard in the Cochrane Handbook for Systematic Review of Interventions V.5.1.0 (updated March 2011).^[30]^ In order to determine the level of risk, the following items would be considered: random sequence, blinding or not, allocation concealment, blinding of outcome assessment, selective reporting, complete or not, other bias. Across the 7 domains, there are 3 grades (“high,” “low,” or “unclear”) to assess the risk of bias of the studies. If we could not judge the study with high or low risk bias because it makes no mention, we regard it unclear risk of bias.

### 2.8. Statistic analysis

The data would be analyzed by using the Stata 15. The enumeration data will be shown as RR and the measurement data will be shown as MD. All the effect sizes will be expressed as 95% confidence intervals.

### 2.9. Assessment of heterogeneity

Heterogeneity will be assessed by *I*^2^ statistics and *χ*^2^ statistics. For purpose of testing the degree of the aberrance among the studies, heterogeneity assessed by *I*^2^ statistics will be used. More than 50% deems to be substantial heterogeneity. If the tests for heterogeneity have no significant meaning (*I*^2^ ≤ 50%), the fixed effect model would be used for data analysis. Otherwise, we would pool and analyze data using a random-effects model.

### 2.10. Assessment of reporting bias

Funnel plots will be performed to evaluate reporting bias. We will use funnel plots to detect potential reporting bias. Begg and Egger test will be used to assess the symmetry of the funnel plot and detect publication bias.

### 2.11. Sensitivity analysis

We will carry out a sensitivity analysis to examine the dependability of the results and check whether there are any particular study leading to an obvious heterogeneity. If yes, we would read over the study and find the reason. If not, that means the results are reliable.

### 2.12. Subgroup analysis

Subgroup analyses will be performed according to the following items: course of the treatment, ages of the patients: children or adults, the original or relative prescription of CLMD.

### 2.13. Quality of evidence

The certainty of evidence will be evaluated by the Grading of Recommendations Assessment, Development, and Evaluation. The following factors will be taken into consideration: limitations in the design, inconsistencies, indirect evidence, hidden error and selective publication of positive results. Evidence quality will be judged as high, moderate, low or very low.

### 2.14. Ethics and dissemination

Our aim is that this systematic review could be published in a peer-reviewed journal. The results will provide evidence regarding the efficacy and safety of CLMD in treating PSI. Participants’ privacy not being involved, this systematic review will not require informed consent form.

### 2.15. Patient and public involvement

No patients or public will be involved.

## 3. Discussion

Insomnia as a common complication after cerebral apoplexy, can easily lead to negative emotions such as anxiety and depression, which is not conducive to the recovery of neurological function, leading to increased mortality and disability rates, and seriously affecting the quality of life. At present, the pathogenesis of PSI has not been clarified, and western medicine treatment plays a general role on PSI, along with more adverse reactions.^[31]^ Western medicine has entered a bottleneck stage. On the other hand, traditional Chinese medicine regards that PSI is mostly caused by emotions and internal injuries of the body, so as to relieve depression and relieve liver. CLMD from Treatise on Febrile Diseases, has the effect of relieving yang and heat, relieving shaoyang, as well as calming the mind and tranquilizing the mind.^[28]^ Clinical studies have reported that CLMD is significant for PSI. This study will systematically review the efficacy and safety of CLMD in the treatment of PSI, providing a new idea for the clinical treatment of PSI.

## Author contributions

**Writing – original draft:** Xuedi Huang, Yue Xiong, Sichen Jiang, Lihua Tang, Xingzhen Lin, Yuzhen Shi, Yaying Xie, Tianzhong Peng

**Writing – review & editing:** Xuedi Huang, Yue Xiong, Sichen Jiang, Lihua Tang, Xinyue Fang, Yuzhen Shi, Wanning Lan, Tianzhong Peng

## References

[1] Ribeiro NF Madruga L. A sudden and severe depressive episode after a left cingulate gyrus stroke: a case report of post-stroke depression and review of literature. J Neural Transm. 2021;128:711–6. 33825944 10.1007/s00702-021-02334-y

[2] Fulk GD Boyne P Hauger M. The impact of sleep disorders on functional recovery and participation following stroke: a systematic review and meta-analysis. Neurorehabil Neural Repair. 2020;34:1050–61. 33153378 10.1177/1545968320962501

[3] Lin TC Zeng BY Chen YW. Cerebrovascular accident risk in a population with periodic limb movements of sleep: a preliminary meta-analysis. Cerebrovasc Dis. 2018;46:1–9. 10.1159/000490065 29982243

[4] American Psychiatric Association. Diagnostic and Statistical Manual of Mental Disorders. 5th ed, Arlington, VA: American Psychiatric Association Publishing; 2013.

[5] Baylan S Griffiths S Grant N. Incidence and prevalence of post-stroke insomnia: a systematic review and meta-analysis. Sleep Med Rev. 2019;49:101222. 31739180 10.1016/j.smrv.2019.101222

[6] Fulk G Duncan P Klingman KJ. Sleep problems worsen health-related quality of life and participation during the first 12 months of stroke rehabilitation. Clin Rehabil. 2020;34:026921552093594. 10.1177/0269215520935940 PMC11145505 32602376

[7] Tang WK Lau CG Mok V. Insomnia and health-related quality of life in stroke. Top Stroke Rehabil. 2015;22:201–7. 25908494 10.1179/1074935714Z.0000000026

[8] Costentin J. Treatment of insomnia. Pharmacological approaches and their limitations. Bull Acad Natl Med. 2011;195:1583–94; discussion 1594. discussion 15945. 22812162

[9] Cai H Wang XP Yang GY. Sleep disorders in stroke: an update on management. Aging Dis. 2021;12:570–85. 33815883 10.14336/AD.2020.0707 PMC7990374

[10] Zhang Y He X Hu S. Efficacy and safety of massage in the treatment of post-stroke insomnia: a protocol for systematic review and meta-analysis. Medicine (Baltimore). 2020;99:e23598. 33371092 10.1097/MD.0000000000023598 PMC7748325

[11] Xiang J Li H Xiong J. Acupuncture for post-stroke insomnia: a protocol for systematic review and meta-analysis. Medicine (Baltimore). 2020;99:e21381. 32791749 10.1097/MD.0000000000021381 PMC7386967

[12] Huang XD Peng TZ Jiang SC. Research progress on pathogenesis and TCM treatment of insomnia after apoplexy. Mod Dist Educ Chin Med. 2021;19:196–9.

[13] Xu WJ Wang F Zhou YX. Preliminary study on etiological mechanism of insomnia after cerebral apoplexy. Shaanxi Tradit Chin Med. 2020;41:134–6.

[14] Lyu D Wang J Yang F. Effect of Tai Chi on post-stroke non-motor disorders: a systematic review and meta-analysis of randomized controlled trials. Clin Rehabil. 2020;35:26026921552095102–38. 10.1177/0269215520951020 32808532

[15] Zhang H Liu P Wu X. Effectiveness of Chinese herbal medicine for patients with primary insomnia: a PRISMA-compliant meta-analysis. Medicine (Madr). 2019;98:e15967. 10.1097/MD.0000000000015967 PMC6587651 31192935

[16] Aulina S Sfha A Bintang AK. Effect of behavioral intervention on the severity of post stroke insomnia. Med Clín Práct. 2021;4.

[17] Xiao M Feng L Wang Q. The therapeutic effects and safety of bright light therapy combined with escitalopram oxalate on insomnia in patients with poststroke depression. Int J Geriatr Psychiatry. 2021;36:182–9. 32830332 10.1002/gps.5412

[18] Aernout E Benradia I Hazo JB. International study of the prevalence and factors associated with insomnia in the general population - ScienceDirect. Sleep Med. 2021;82:186–92. 33957414 10.1016/j.sleep.2021.03.028

[19] Sharma M Patel V Acharya UR. Automated identification of insomnia using optimal bi-orthogonal wavelet transform technique with single-channel EEG signals. Knowl Based Syst. 2021;224:107078–83.

[20] Thakral M Korff MV Mccurry SM. ISI-3: evaluation of a brief screening tool for insomnia. Sleep Med. 2021;82:104–9. 33910159 10.1016/j.sleep.2020.08.027 PMC8141095

[21] Xia S Yang P Li F. Chaihu-Longgu-Muli decoction exerts an antiepileptic effect in rats by improving pyroptosis in hippocampal neurons. J Ethnopharmacol. 2021;270:113794. 33422654 10.1016/j.jep.2021.113794

[22] Zhang XY Huang NN Sun KB. Efficacy network and mechanism prediction of Chaihu Longgu Muli decoction in treatment of essential hypertension. Chin Tradit Herbal Drugs. 2019:5162–9.

[23] Scheid V Bensky D Ellis A. Chinese Herbal Medicine: Formulas & Strategies. Washington: Eastland Press; 1990:387

[24] Wang X Ju J Li J. Chaihu Longgu Muli Decoction, a Chinese herbal formula, for the treatment of insomnia: a systematic review and meta-analysis. Medicine (Baltimore). 2020;99:e22462. 33019437 10.1097/MD.0000000000022462 PMC7535661

[25] Zhang SM. Clinical Study of Insomnia After Stroke and Meta-Analysis of TCM Treatment. Guangzhou University of Chinese Medicine; 2020.

[26] Tang R. Randomized parallel controlled study on the treatment of post stroke insomnia with Bupleurum Chinense plus oyster shell decoction. J Pract Tradit Chin Intern Med. 2017;31:17–20.

[27] Wang PQ Yin WU Zhang M. Analysis of protective effects of Chaihu Jia Longgu Muli Tang against ischemic stroke by combining traditional Chinese medicine pathogenesis and efficacy with modern pathology and pharmacology. China J Chin Mater Med. 2018;43:2448–53. 10.19540/j.cnki.cjcmm.2018.0076 29950058

[28] Li X Sun XL. Effects of Bupleurum and Longguoyster decoction on sleep quality, serum 5-HT, and DA in patients with insomnia after stroke. Electron J Mod Med Health Res. 2020;4:131–132, 134.

[29] Bassetti C Randerath W Vignatelli L. EAN/ERS/ESO/ESRS statement on the impact of sleep disorders on risk and outcome of stroke. Eur J Neurol. 2020;27:1117–36. 32314498 10.1111/ene.14201

[30] Piram M Mahr A. Epidemiology of immunoglobulin A vasculitis (Henoch-Schönlein): current state of knowledge. Curr Opin Rheumatol. 2013;25:171–8. 23318735 10.1097/BOR.0b013e32835d8e2a

[31] Fu XB. Clinical efficacy of Bupleurum and Longguoyster Decoction in the treatment of insomnia after stroke. Med Equip. 2019;32:115–6.

